# Effect of MWCNTs on Gastric Emptying in Mice

**DOI:** 10.1007/s11671-010-9803-y

**Published:** 2010-10-07

**Authors:** Z Li, W Qi, YX Geng, DQ Pan, Y Lu, JZ Xu, WS Wu

**Affiliations:** 1 Radiochemistry Laboratory, Lanzhou University, 73000, Lanzhou, People's Republic of China

**Keywords:** oMWCNTs, Functional disorder, NO, Ach, Gastric emptying

## Abstract

After making model of gastric functional disorder (FD), part of model mice were injected intravenously (i.v.) with oxide multi-walled carbon nanotubes (oMWCNTs) to investigate effect of carbon nanotubes on gastric emptying. The results showed that NO content in stomach, compared with model group, was decreased significantly and close to normal level post-injection with oMWCNTs (500 and 800 μg/mouse). In contrast to FD or normal groups, the content of acetylcholine (Ach) in stomach was increased obviously in injection group with 500 or 800 μg/mouse of oMWCNTs. The kinetic curve of emptying was fitted to calculate gastric motility factor *k*; the results showed that the *k* of injection group was much higher than FD and normal. In other words, the gastric motility of FD mice was enhanced via injection with oMWCNTs. In certain dosage, oMWCNTs could improve gastric emptying and motility.

## Introduction

Carbon nanotubes (CNT) represent the structural evolution of the archetypal molecular architecture consisting of pure carbon units, the C_60_ fullerene [1], CNT, or "buckytubes" [2], possess extraordinary properties, such as high electrical and thermal conductivity, great strength, and rigidity, and being developed for a wealth of applications, including field emission [3], energy storage, molecular electronics, and atomic force microscopy. These properties indicate diverse future biomedical uses in areas such as targeted chemotherapeutics, in vitro cell markers, diagnostic imaging contrast agents, biochemical sensors, and photoablative therapy agents [4]. However, it was not found in previous works to use carbon nanotubes as single drug to therapy in clinical application, no works could prove that the carbon nanotubes can improve biological function of animals, and most papers only pay attention to toxicology of carbon nanotubes on tissues or cell [5-12]. In our long-term researches, we found that the oxide multi-walled carbon nanotubes (oMWCNTs) can improve gastric function of animals. Therefore, we attempt to investigate effect of oMWCNTs on gastric emptying in mice. If the CNTs alone have medicinal values, then it is very important for CNTs to develop in clinical application. It would be inevitable to widen the application prospects of CNTs in medical field.

## Materials and Method

### Preparation of Oxidized MWCNTs

MWCNTs commercially prepared by chemical vaporization deposition were obtained from Shenzhen Nanotech Port Co. Ltd. China. Determined with transmission electron microscopy (TEM), MWCNTs are several tens of micrometers in length, with a diameter of 10–30 nm. Purity was >96%, containing <3% amorphous carbon and ash < 0.2 wt%, according to thermal gravity analysis (TGA).

The as-grown MWCNTs (named as untreated MWCNTs) were added into the solution of 3 mol/L HNO_3_ to remove the hemispherical caps of the nanotubes. The mixture of 3 g MWCNTs and 400 mL 3 mol/L HNO_3_ was ultrasonically stirred for 24 h. The suspension was filtrated, and then dialyzed by dialysis bag for 2 weeks, and rinsed with deionized water until the pH of the suspension reached about 6, and then was dried at 80°C. Thus, prepared MWCNTs (named as oxidized MWCNTs or oMWCNTs) were calcined at 450°C for 24 h to remove the amorphous carbon [13]. The oMWCNTs were dissolved in normal saline, and then ultrasonically treated before injection.

### Preparation of the Test Meal

Hydrated diet was prepared by placing 45 g of pellets in 100 ml water and storing in the refrigerator at 4°C for at least 16 h. Before dispensing the food to the animals, a further 2 mL water was added to ensure that the food was saturated with water yet maintained a semi-solid state. The hydrated diet was initially introduced because it was observed that in mice fed on standard dry chow, gastric emptying was very slow. The weight of residual food in the stomach of the animals following 24 h of food deprivation was still very substantial, even with a grid floor in place, and this made evaluation of gastric emptying difficult [14,15].

### Test of NO and ChAT

Female Kongming White mice weighing 16–20 g were obtained from laboratory Center for Medical Science, Lanzhou University, Gansu, China. All animals were introduced to hydrated diet (prepared as described above) 24–36 h before commencing the experiment. The animals were maintained on the hydrated diet for 24–36 h prior to commencement of the experiments in order to allow them to adapt to the new food. Water was provided ad libitum throughout the experimental period.

The four groups of mice (fifteen mice per group) were injected intraperitoneally (i.p.) with L-arginine [16] (6 mg/mouse) for 5 days to make model of FD. One additional group (fifteen mice) was injected intraperitoneally with normal saline as control. Until at 5 day, three groups of FD were injected intravenously (i.v.) with oMWCNTs for 3 days, the doses were 100, 500, and 800 μg/mouse, respectively. Another one was continuing to inject with L-arginine for 3 days. All mice were killed at 8 days; every stomach was collected, and then removed chyme. According to the procedures of specification, the ChAT and NO kits, purchased from Nanjing Jiancheng Bio-Technology Co., Ltd., were used to determine content of ChAT and NO in stomach tissues.

### The Effect of oMWCNTs on the Secretion of Gastric Mucus [17] and the Activity of Pepsin

After making successful model of FD, one group of FD (fifteen mice) was injected intravenously with oMWCNTs (500 μg/mouse) for 3 days, the other group of FD was injected intraperitoneally with L-arginine (6 mg/mouse) for 3 days. Meanwhile, one normal group (fifteen mice) was injected intraperitoneally with normal saline as control. All mice were killed at 1 h after fed with hydrated diet to collect chyme and gastric tissues, and then the gastric mucosa was washed by 4 mL water, the flushing fluid was used to dissolve 1 g chyme. Suspension of 0.25 g/mL (solid–liquid ratio, S/L) was soaked for 24 h and centrifuged to measure pH values in supernatants. Meanwhile, the pepsin kits, purchased from Nanjing Jiancheng Bio-Technology Co., Ltd., were used to determine the pepsin activity in gastric tissues.

### Kinetic Studies

Two groups, 42 mice/per group, were used to make FD model according to the above methods, and another group of 42 mice as control was treated with normal saline. All mice were fed with hydrated diet (prepared as described above) for 5 days at 9:00, and water was provided ad libitum throughout the experimental period. All animals were allowed equal access to it for 1 h (free-feeding period). After successful modeling, mice of group one were injected intravenously with oMWCNTs for 3 days (500 μg/mouse), and the other one were continuing to inject intraperitoneally with L-arginine for 3 days (6 mg/mouse). Until day 8, after injection with oMWCNTs, L-arginine or normal saline, all mice (including control) were fed for 1 h with above food. Subsequently, 42 mice with 8 in each group were, respectively, killed at 0, 0.5, 1, 2, 4, and 6 h; total stomach of each mouse were collected and weighed, and then the chyme was removed and cleaned with normal saline, net gastric tissues were weighed. The emptying rate was calculated according to following formulae:

$$E=\frac{W_{\text{st}}-W_{\text{sn}}}{W_{\text{b}}}\times 100\%$$

*E* emptying rate, *W*_st_ weighs of total stomach, *W*_sn_ weighs of net stomach, *W*_b_ body weighs.

### Analysis of Data

The data were expressed as mean ± SEM, and statistical significance of differences was calculated using SPSS17.0 software to perform one-way ANOVA test.

## Results and Discussion

### Preparation of oMWCNTs

**Figure 1:**
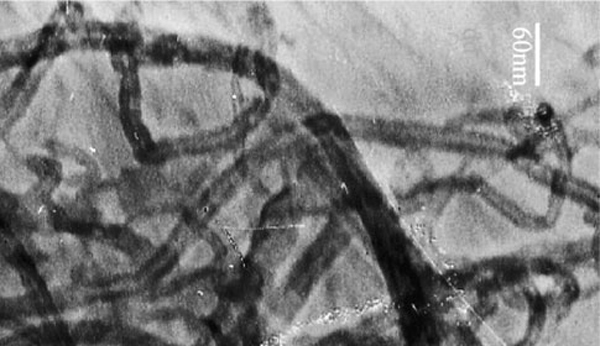
**TEM of oxide multi-walled carbon nanotubes**.

**Figure 2:**
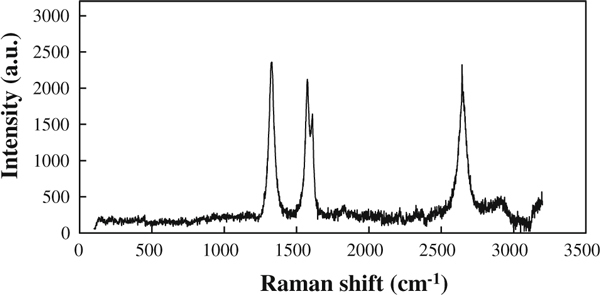
**Raman spectra of oxide multi-walled carbon nanotubes**.

The transmission electron microscopy (TEM) of oMWCNTs was shown in Figure 1. oMWCNTs were characterized only by Raman spectra (Figure 2), the peak at 1,324 cm^-1^ was assigned to the D line, and the peak at 1,570 cm^-1^ was assigned to the G line [18].

### Distribution of oMWCNTs in Stomach and Chyme

**Figure 3:**
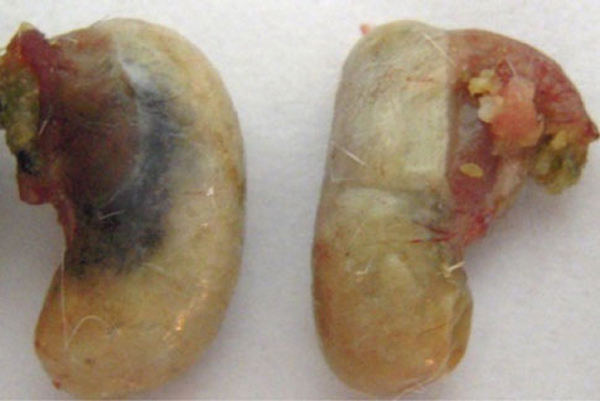
**Distribution of oMWCNTs in stomach post-i.v. with 800 μg/mouse**. Left is injection group, right is control group.

**Figure 4:**
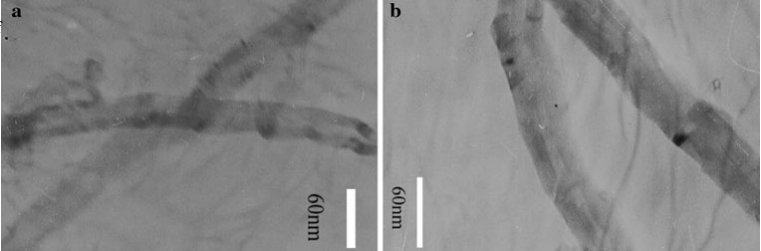
**a TEM of oMWCNTs in gastric tissues and chyme**. **b** TEM of oMWCNTs in chyme.

The black nanoparticles were located in the lesser curvature of the stomach (Figure 3) post-i.v. injection with 800 μg/mouse oMWCNTs. The oMWCNTs were also detected in chyme by TEM (Figure 4). Therefore, the oMWCNTs could pass through stomach tissues into chyme post-i.v. injection. As observed in Figure 3, the nanomaterials were located in the lesser curvature of the stomach, which placed them adjacent to the pyloric gland. The pyloric gland secretes mucus and bicarbonate (HCO_3_^-^) to form a mucus bicarbonate barrier. This HCO_3_^-^ is produced by dissociation of H_2_CO_3_, which is generated from CO_2_ and H_2_O from blood via carbonic anhydrase (CA) catalysis in stomach mucosa oxyntic cells. Some of the HCO_3_^-^ can then cross the intercellular space into mucus cells of the pyloric gland and be secreted into chyme [19]. oMWCNTs contain large numbers of –COOH and –OH groups, and we speculate that X-(COOH)_*n*_ (X: oMWCNTs) would decompose to X-(COO^-^)_*n*_ and *n*H^+^ in the intercellular space. Part of the X-(COO^-^)_*n*_ could then enter into blood, and part could diffuse across the cellular membrane into mucus cells. It could then be secreted as part of the mucus bicarbonate barrier and enter chyme. Because oMWCNTs were secreted from mucus cells into chyme, and the H^+^ that could be secreted stably from parietal cells [20] was not affected by the secretion of mucus, the total pH in stomach would be determined by alkali content in gastric mucus. Larger numbers of oMWCNTs-(COO^-^)_*n*_ secreted from gastric mucus cells would affect and change gastric pH in administration groups. So the effect of oMWCNTs on the secretion of gastric mucus could be investigated by detecting pH changes in stomach [17].

### The Effect of oMWCNTs on the Secretion of Gastric Mucus

In contrast to normal group, the pH in stomach was not affected by L-arginine in FD group (*p* > 0.05), but pH was increased obviously in administration group compared with FD or normal group (*p* < 0.05, Table 1). As can be known from frontal discussion, oMWCNTs-(COO^-^)_*n*_ could be secreted from gastric mucus cells as part of mucus bicarbonate barrier. Therefore, the part of gastric acid (H^+^) would be neutralized by oMWCNTs-(COO^-^)_*n*_ in mucus and increase pH in stomach (Table 1), which verified our frontal secretion hypothesis of oMWCNTs.

**Table 1:** The effect of oMWCNTs on pH in stomach

Groups	FD	oMWCNTs	Normal
pH	4.69 ± 0.04	4.75 ± 0.04*^,&^	4.68 ± 0.06

* *p* < 0.05, groups versus normal; ^&^*p* < 0.05, groups versus FD

### Test of NO and ChAT in Stomach

**Figure 5:**
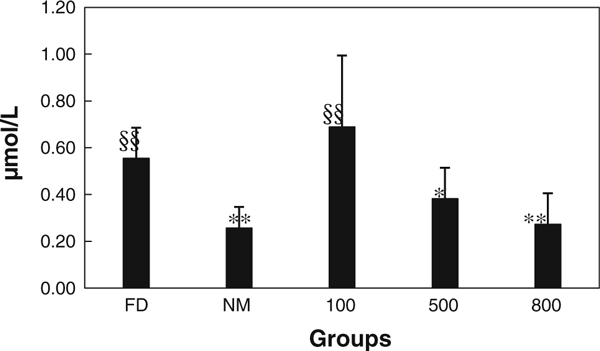
**Content of NO in stomach from FD, normal, 100, 500, and 800 μg groups**.

**Figure 6:**
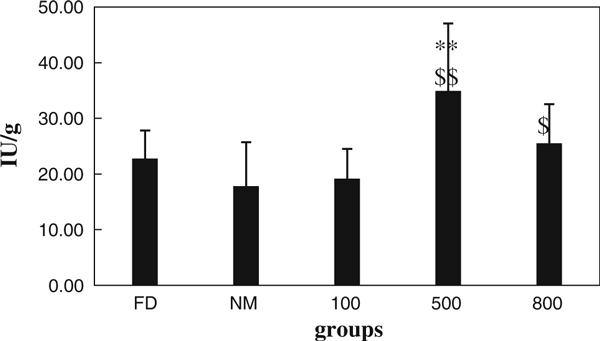
**Content of ChAT in stomach from FD, normal, 100, 500, and 800 μg groups**.

The content of NO and ChAT has been shown in Figures 5 and 6. The results indicated that obvious difference has been seen post-i.v. with different dose of oMWCNTs. For group model, the content NO compared with normal has been decreased in stomach (*p* < 0.01). After injection with 100 μg/mouse of oMWCNTs, the content of NO and ChAT was no change compared with FD (*p* > 0.05). However, for injection with 500 μg/mouse of oMWCNTs, content of NO has been significantly decreased compared with FD (0.01 <*p*<0.05), but on change compared with normal (*p* > 0.05); the content of ChAT has been increased significantly compared with normal and FD post-i.v. with 500 μg/mouse(*p* < 0.01). Meanwhile, after i.v. with 800 μg/mouse, it also could induce significant decreasing of NO in stomach compared with FD(*p* < 0.01); the content of ChAT was increased post-i.v. with 800 μg/mouse compared with normal(0.01 <*p*<0.05), but no change compared with FD (*p* > 0.05).

The choline acetyl transferase (ChAt) was synthase of acetylcholine (Ach), which indicated that ChAt content could represent Ach content in stomach; previous studies showed that gastric active function is complex physical process, which was regulated by body fluid and nerve [21]. The coordination of excitatory and inhibitory neuron in midgut never regulates the gastrointestinal coordinated motion. The neurotransmitter released from excitatory neuron is Ach, which could promote contraction of gastrointestinal smooth muscle. The neurotransmitter released from inhibitory neuron is NO, which could induce relaxation of gastrointestinal smooth muscle. NO produced by nitric oxide synthase is the neurotransmitter of non-adrenergic and non-cholinergic nerves. The NO could promote the capacity relaxation of stomach and antagonize contraction of stomach induced by ChAt. In a word, the NO could affect on gastric peristalsis and emptying [21].

The L-arginine could induce synthesis of NO from nitric oxide synthase [16]. The results showed that L-arginine has promoted increasing of NO in stomach of normal mice, so model of FD was made successfully for 5 days post-i.p. with L-arginine and that reported by literature [16]. Lower dose of injection with oMWCNTs (100 μg/mouse) did not induce changes of NO and ChAT in stomach, but obvious effect has been observed post-i.v. with 500 or 800 μg/mouse (*p* < 0.01, Figures 5, 6). The higher content Ach could facilitate contraction of gastrointestinal smooth muscle, and lower content NO could inhibit relaxation of gastrointestinal smooth muscle [16,21]. Therefore, the emptying force of stomach has been improved post-i.v. with higher dose of oMWCNTs. This implied that the gastric emptying could be enforced significantly after i.v. with higher dose of oMWCNTs (500, 800 μg/mouse).

### Effect of oMWCNTs on the Secretion of Pepsin in Stomach

**Figure 7:**
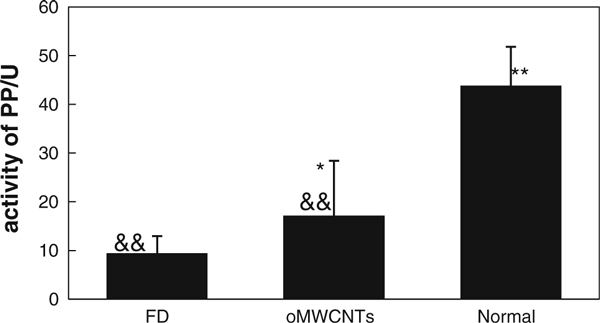
**The effect of oMWCNTs on the pepsin activity**. * *p* < 0.05; ** *p* < 0.01 groups versus FD; ^&&^*p* < 0.01 groups versus normal.

Figure 7 showed that L-arginine could decrease significantly the activity of pepsin in FD group (*p* < 0.01) compared with normal mice; the activity of pepsin was increased (*p* < 0.05) in administration group compared with FD group after i.v. with oMWCNTs of 500 μg/per mouse, but still lower much than normal level (*p* < 0.01).

The pepsinogen that was secreted from gastric chief cells could be activated and transformed into pepsin in pH < 5.0, the pepsin could decompose the protein of chyme [20]. Therefore, the activity of pepsin could be increased by improving the secretion of pepsinogen under stable pH values. Figure 7 showed that L-arginine could decrease significantly the activity of pepsin in FD group (*p* < 0.01) compared with normal mice, it was reported that L-arginine could increase NO content [16] so as to induce gastric functional disorder [21], but could not directly inhibit the secretion of pepsinogen, so these results implied that gastric functional disorder caused by L-arginine could inhibit the secretion of pepsinogen and decrease the pepsin activity in FD group. Hereby, we concluded that the secretion of pepsinogen in administration group could be increased slightly because of gastric functional disorder has been improved *via* injection with oMWCNTs. Therefore, the pepsin activity was increased to some extent in administration group due to the secretion of pepsinogen improved by carbon nanotubes (Figure 7).

### Kinetic Study of Gastric Emptying

The force of gastric emptying originated from the difference between capacity relaxation pressures of gastric fundus and duodenal pressures with liquid food fed [22]. So the stomach could be equivalent to an elastic peltry, and then the capacity relaxation pressure would be seen as an elastic force, so which could be related to gastric distension. This meant the more food residue in stomach, the stronger elastic force, and the quicker speed of emptying. As a result, if the *v* is speed of emptying, then:

(1) $$v=-\frac{\text{d}W}{\text{d}t}=kW.$$

*W* is weighs of food residue, *W* = *W*_st_ - *W*_sn_ (*W*_st_ is total weighs of stomach, *W*_sn_ is net weighs of stomach); *t* is time; *k* gastric motility factor (equivalent to elastic coefficient); the *k* is related to gastric function and food state (liquid in here); when the same food was fed, the lager *k* is, the stronger emptying force is.

Deduction of formula (1)

(2) $$W=e^{-kt}=W_{\text{st}}-W_{\text{sn}}$$

(3) $$\frac{W_{\text{st}}-W_{\text{sn}}}{W_{\text{b}}}=\frac{1}{W_{\text{b}}}e^{-kt}=E;\;\text{if}\;\frac{1}{W_{\text{b}}}=A;\;\text{so}\;E=Ae^{-kt}$$

taken logarithm:

(4) lnE=lnA−kt.

**Figure 8:**
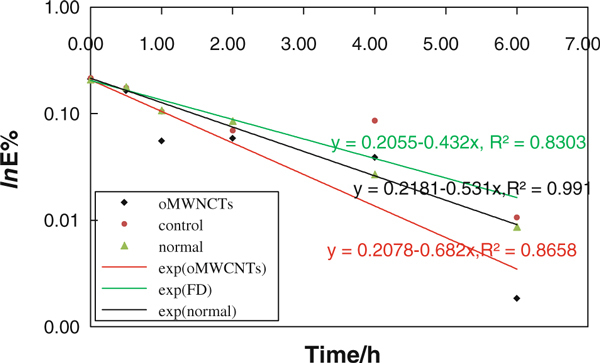
**The fitting curve of gastric emptying in FD, normal and 500 μg of oMWCNTs**.

The formula (4) showed linear dependence between the *ln E* and *t*. The *A* was related to body weighs. This model could be called as peltry model (PM) in our paper. It was reported that the emptying curve of liquid food was in accord with single exponential model [23], but curve of solid food digested easily was close to linear type [24]. In our experiment, the flowing hydrated diet was used, so the curve should be in accord with single exponential model (Figure 8).

**Figure 9:**
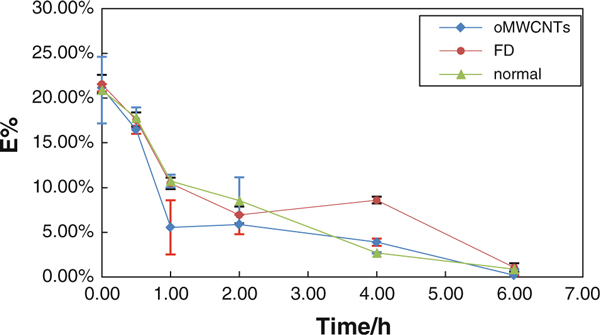
**Kinetic curve of gastric emptying in FD, normal and 500 μg of oMWCNTs**.

Our PM was in agreement with that reported in the work of Gaudichon et al. [24], and the fitting of curve according to PM was very good in normal mice fed with semi-fluid food, the fitting coefficient *R*^2^ was 0.991(Figure 8). The results indicated that the PM could simulate factually the emptying kinetic change of semi-fluid food in stomach. But for FD and injection group, the fitting degree was poorer; *R*^2^ of them was 0.8303 and 0.8658, respectively (Figure 8). The *E*% was rapidly decreased from 0 to 1 h in injection group with 500 μg/mouse and decreased to ~5% at 1 h; the speed of decreasing was slow from 1 to 6 h (Figure 9). The decreasing speed of *E*% in FD group was similar to the normal group before 1 h, but the decreasing of *E*% was very slow from 1 to 4 h (10.47–8.86%); after 4 h, it was decreased rapidly to ~0% at 6 h (Figure 9).

Figures 3 and 8 showed that the emptying force of injection group should be higher than FD and normal because of higher Ach and lower NO content in stomach, but the emptying force of FD was lower than normal mice for normal Ach and high-NO content in stomach. So the food was emptied rapidly from stomach in 1 h for injection groups, but for FD, slow emptying would be observed from 1 to 6 h for poor gastric emptying force.

According to above PM, gastric motility factor *k* was fitted to calculate in FD, normal and injection groups (Figure 8). When the same food was used in experiment, then the higher the *k* is, the stronger the gastric emptying is and the better the gastric function is. Figure 8 showed that the *k* of injection group with 500 μg/mouse was much higher than normal group and FD, the *k* of injection, FD and normal were 0.682, 0.531 and 0.432, respectively. It implied that oMWCNTs could improve gastric function of FD and enhance gastric motility and emptying.

As can be seen from Figures 3 and 4 and Table 1, the oMWCNTs could be accumulated in stomach and secreted into chyme as mucus bicarbonate barrier, so in the course of accumulation and secretion, oMWCNTs had to contact with gastric mucous, as one kind of foreign body, stimulated gastric tissues, caused a series of reactions, and decreased NO content and increased ChAT content in gastric tissues, sped up the gastric emptying, and improved to some extent the activity of pepsin. As a result, the physiological function of stomach was improved obviously post-i.v. with suitable dose of oMWCNTs. And we concluded that the high distribution of oMWCNTs in stomach was result from oxide treatment, so surface chemical groups on carbon nanotubes would be a key factor to affect on gastric physiological function. Therefore, after oxide treatment, the CNTs, injected into mice of functional disorder, could inhibit content NO and increase the content Ach in stomach, and it was more favorable for higher dose of CNTs to reinforce the effect.

## Conclusion

1. The oMWCNTs can be secreted from mucus cells into chyme post-i.v. with oMWCNTs, and this course can increase pH in stomach.

2. The NO content can be decreased post-i.v. with carbon nanotubes into mice of functional disorder, and the Ach content can be increased, the effect is more obvious post-injection with higher dose of carbon nanotubes.

3. The carbon nanotubes can enhance gastric emptying and improve gastric function, and thus increase to some extent the activity of gastric pepsin.
